# Tips for assessing vision in a baby or child

**Published:** 2019-12-17

**Authors:** Richard Bowman

**Affiliations:** 1 Honorary Clinical Consultant: International Centre for Eye Health, London School of Hygiene and Tropical Medicine, London, UK.

**In babies and young children, early intervention can prevent decades of visual impairment. This article gives tips and advice for a successful eye examination and explains what responses to expect from a healthy baby.**

**Figure:**
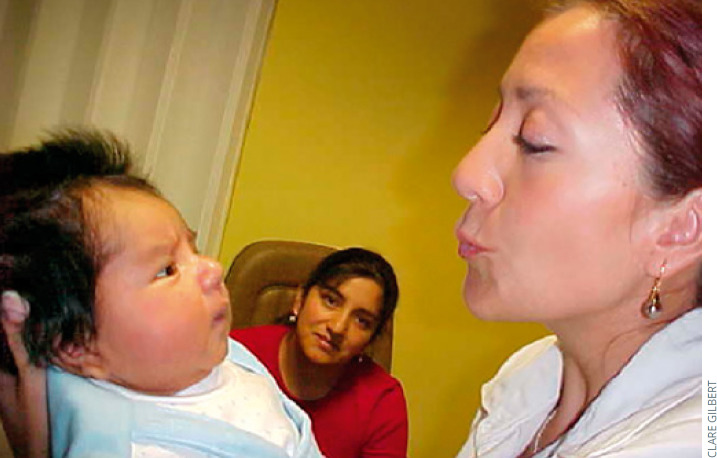
An eye care worker checks a baby's fixation. The baby is looking at her face, which is a reassuring sign.

## Assessing vision in a baby (0–1 year)

There is no need to be anxious about examining a baby. If the baby is awake and attentive, there is a lot you can find out by asking the parents and simply observing the baby's reactions.

- First ask the parents what they think about their baby's vision.
- Notice how the baby looks at things in the room, such as the window or any lights.
- Watch for eye contact between the baby and parents.
- Does the baby look when someone comes into the room?
- Does the baby respond to silent smiles or to raised eyebrows?
- Do you get eye contact?

**Retinoblastoma** is an extremely rare form of cancer that affects babies and very young children. Early detection, using the ‘red’ reflex test (p. 54), can save a child's life. Note: a healthy reflex looks paler in someone with darker skin and a pigmented fundus.

You should have realistic expectations about what a baby should be able to do by a certain age. Table 1 shows when a baby is too young to show a visual response, when the response is likely to develop, and at what age you should be worried if a baby does NOT show the expected response. You can ask the mother or check the baby's responses yourself.

For example, if a baby of about three weeks old does not turn to a diffuse light, such as light coming from a window, you would not necessarily be worried – although you would still believe the parents if they are concerned. On the other hand, if a baby is eight weeks old and does not eventually turn to a diffuse light, then there may be a problem and you should investigate further.

Bear in mind that there can be a lot of variation in babies' development; however, this table should be a helpful guide.

**Table 1:** Normal visual functioning for a baby

Behaviour	Age Neonate	Age 6 weeks	Age 3 months	Age 4 months	Age 5 months +
Blinks when a light is flashed in their eyes?	Healthy babies will do this. If not, suspect a problem				
Turns to a diffuse light, such a light coming from a window?	May do it	Healthy babies will do this. If not, suspect a problem			
Looks at your face when 10–20 cm away (less than 1 foot)? Any response to silent smiles or eyebrow raising?	Too young	May do it	Healthy babies will do this. If not, suspect a problem		
Eyes fix on, and follow, a dangling ball or toy?	Too young	May do it	Healthy babies will do this. If not, suspect a problem		
Watches an adult at 1.5 metres (5 feet)?	Too young	May do it		Healthy babies will do this. If not, suspect a problem	
Converges accurately? (If you move a toy closer and further away, do the eyes focus on the toy and line up properly?)	Too young	May do it		Healthy babies will do this. If not, suspect a problem	
Blinks in response to a threat? (Any silent, sudden movement close to the face which causes no breeze, e.g., opening your fist very suddenly.)	Too young	Too young	Too young	May do it	Healthy babies will do this. If not, suspect a problem

## Tips for examining a baby

**Figure 1:**
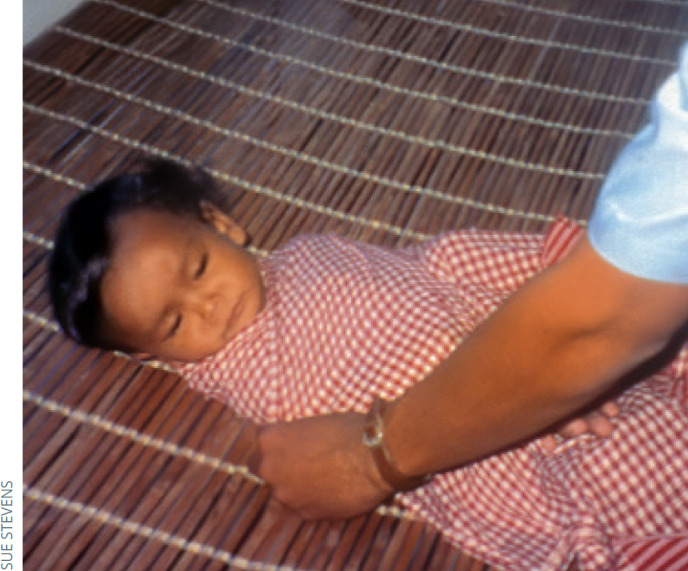
Wrapping a baby for an eye examination. This baby found it very comforting and fell asleep!

- Try to carry out as much of the examination as possible without touching the baby. Children often resist having their eyes held open, for example.
- Have many toys available. For each new toy, the baby will momentarily hold their eyes steady, allowing a quick examination. If available, use toys which are bright and can flash on and off. A good rule to remember is: “one toy, one look”, as babies can quickly lose interest.
- Don't be embarrassed about making funny noises! These help to attract the baby's attention and keep them interested and calm. Look for good fixation, e.g. on your face.
- In order to perform a more detailed examination of an infant, examine the child while she or he is being bottle fed or breast fed.
- If you are struggling, ask the parent's permission to wrap the baby; the pressure can help babies to feel safe and secure while keeping their hands away from your equipment! To do this, place the baby on a blanket or sheet, hold the arms to the side and the legs straight, and wrap the blanket around the body and arms (Figure 1). Ask the parent to hold the baby. Ask the parent, or a helper, to open one eye at a time by placing a finger very gently on the upper eyelid and easing it upwards; first demonstrate how to do this, using your own eye. Praise and reassure the parents – this may be a very stressful experience for them and their child.

## Assessing vision in a young child (1–5 years)

Children in this age group should have steady eyes, no squint (p. 55), no history of sight difficulties and, if in a good mood, show interest in colourful or interesting objects in the room. They should respond to silent smiles, eyebrow raising, and winking.

Children in this age group should also be able to see objects presented in their peripheral visual field by a colleague while you draw their attention to your face, perhaps by making a funny noise. Cover one eye at a time if the child will allow it and ask them to identify different sized objects or, with older children, letters. Make it a game.

**Figure 2:**
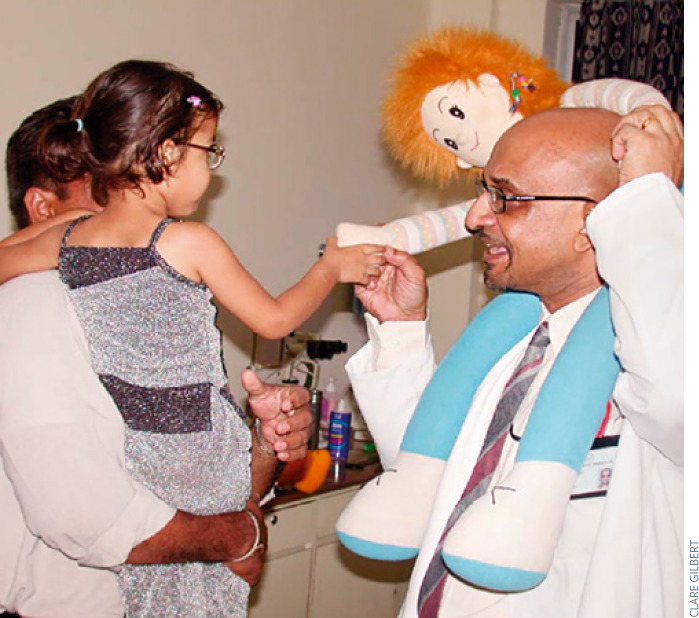
Be playful and make a game of the examination

Many children can accurately name colours by the age of three, but many cannot do this until they are older; it is reassuring if they can.

After the age of three, most children can participate in accurate visual acuity, visual field, and colour vision testing when done by someone trained and with age-appropriate equipment.

If you do not have that equipment, or have not been trained to use it, you can still test a child's functional vision using everyday objects, as described above.

**“Observe children when they don't know they are being observed, for example while you are talking to the mother or taking a history.”**

## Tips for examining a young child

The tips for examining a baby (above) apply equally well to young children.

In addition:

- Be playful and make a game of the examination (Figure 2). For example, shine a light into the mother's eye first, or pretend you are playing ‘hide and seek’ or ‘peekaboo’ when covering one eye.
- Observe children when they don't know they are being observed, for example while you are talking to the mother or taking a history.
- The tip about wrapping up a baby will work for a younger child, but it may be more difficult in an older child. If examination is proving difficult, ask the parents what they think would be appropriate or would work best. For example, parents could hold the child on their lap and wrap their arms around their child in a hug, thereby gently restraining the child's arms.
